# Measurement of Biomolecular Diffusion in Extracellular Matrix Condensed by Fibroblasts Using Fluorescence Correlation Spectroscopy

**DOI:** 10.1371/journal.pone.0082382

**Published:** 2013-11-28

**Authors:** Takanori Kihara, Junri Ito, Jun Miyake

**Affiliations:** 1 Department of Life and Environment Engineering, Faculty of Environmental Engineering, The University of Kitakyushu, 1-1 Hibikino, Wakamatsu, Kitakyushu, Fukuoka, Japan; 2 Department of Mechanical Science and Bioengineering, Graduate School of Engineering Science, Osaka University, 1-3 Machikaneyama, Toyonaka, Osaka, Japan; Osaka University, Japan

## Abstract

The extracellular matrix (ECM) comprises the heterogeneous environment outside of cells in a biological system. The ECM is dynamically organized and regulated, and many biomolecules secreted from cells diffuse throughout the ECM, regulating a variety of cellular processes. Therefore, investigation of the diffusive behaviors of biomolecules in the extracellular environment is critical. In this study, we investigated the diffusion coefficients of biomolecules of various sizes, measuring from 1 to 10 nm in radius, by fluorescence correlation spectroscopy in contracted collagen gel caused by fibroblasts, a traditional culture model of dynamic rearrangement of collagen fibers. The diffusion coefficients of the biomolecules in control collagen gel without cells decreased slightly as compared to those in solution, while the diffusion coefficients of biomolecules in the contracted gel at the cell vicinity decreased dramatically. Additionally, the diffusion coefficients of biomolecules were inversely correlated with molecular radius. In collagen gels populated with fibroblasts, the diffusion coefficient at the cell vicinity clearly decreased in the first 24 h of culture. Furthermore, molecular diffusion was greatly restricted, with a central focus on the populated cells. By using the obtained diffusion coefficients of biomolecules, we calculated the collagen fiber condensation ratio by fibroblasts in the cell vicinity at 3 days of culture to represent a 52-fold concentration. Thus, biomolecular diffusion is restricted in the vicinity of the cells where collagen fibers are highly condensed.

## Introduction

Biological systems consist of heterogeneous components arranged into diverse environments required for sustaining life. For example, the membrane, cytoplasm, nucleoplasm, and extracellular matrix (ECM) are typical heterogeneous environments in biological systems and affect the physicochemical behaviors of biomolecules, which diffuse anomalously throughout these inhomogeneous environments [1]–[3]. The diffusive behaviors of signaling molecules play crucial roles in determining the activity of cells [4]–[6]. In particular, cytokines, ions, and low-molecular-weight compounds secreted from cells diffuse throughout the ECM and regulate target cell fates by affecting paracrine and autocrine signaling [7]–[10]. Therefore, it is important to investigate the diffusion behaviors of molecules in the extracellular environment.

The major components of the ECM are collagens, which make up the intricate, solid fibers in connective tissues, allowing for the construction of the complex, heterogeneous physical environment of the ECM. Type I collagen also forms fibers under physiological conditions *in vitro* and can form gels in which cells can be grown, mimicking a 3-dimensional environment. Collagen gels containing fibroblasts contract, and fibroblasts grown in collagen gel show distinct morphologies and functions from those of cells cultured on 2-dimensional rigid surfaces [11], [12]. Fibroblasts contracting collagen gels are especially used in artificial dermis for regenerative medicine [11]. Cells adhere to collagen fibers via surface receptors and reorganize the alignment of the fibers by actomyosin contraction [13]–[15]. During this process, the collagen fibers are condensed toward the cells, and then the collagen gel contracts [13]. The density of the fully contracted collagen gel has been shown to reach about 15 mg/mL [16], [17]. Thus, contracted collagen gels are considered as *in vitro* models of the rearrangement of collagen fibers and construction of extracellular environment by cells [18]. By examining the diffusion coefficients of molecules in the contracted gel, we can study the physical environment of the heterogeneous ECM constructed by cells.

The diffusion coefficient (*D*) of a molecule can be determined by various spectroscopic techniques used to observe the diffusion behavior of the molecule. Among these spectroscopic techniques, fluorescence correlation spectroscopy (FCS) is an efficient method for the measurement of diffusion coefficients and is frequently applied to various biological systems using confocal laser scanning microscopy (CLSM) [19]. FCS instruments have an extremely small sampling space (the confocal volume is about 0.2–1 fL), and *D* is determined from the time correlation function obtained from the intensity fluctuation of emission from a small number of fluorescent molecules [20]. The sampling space specificity and low concentration of fluorescent molecules are advantages when studying biological samples. Using FCS, the diffusion coefficients of many types of biomolecules in intracellular and extracellular spaces can be determined [1], [21]–[24]. Furthermore, Masuda et al. described the anomalous diffusion behaviors of a low-molecular-weight molecule (Alexa Fluor 488) in 0.1–0.9 wt% of hyaluronan aqueous solution using sampling volume-controlled FCS [3]. Therefore, FCS is an appropriate method to determine the diffusion behavior of many types of biomolecules in solution-filled biological samples.

**Figure 1:**
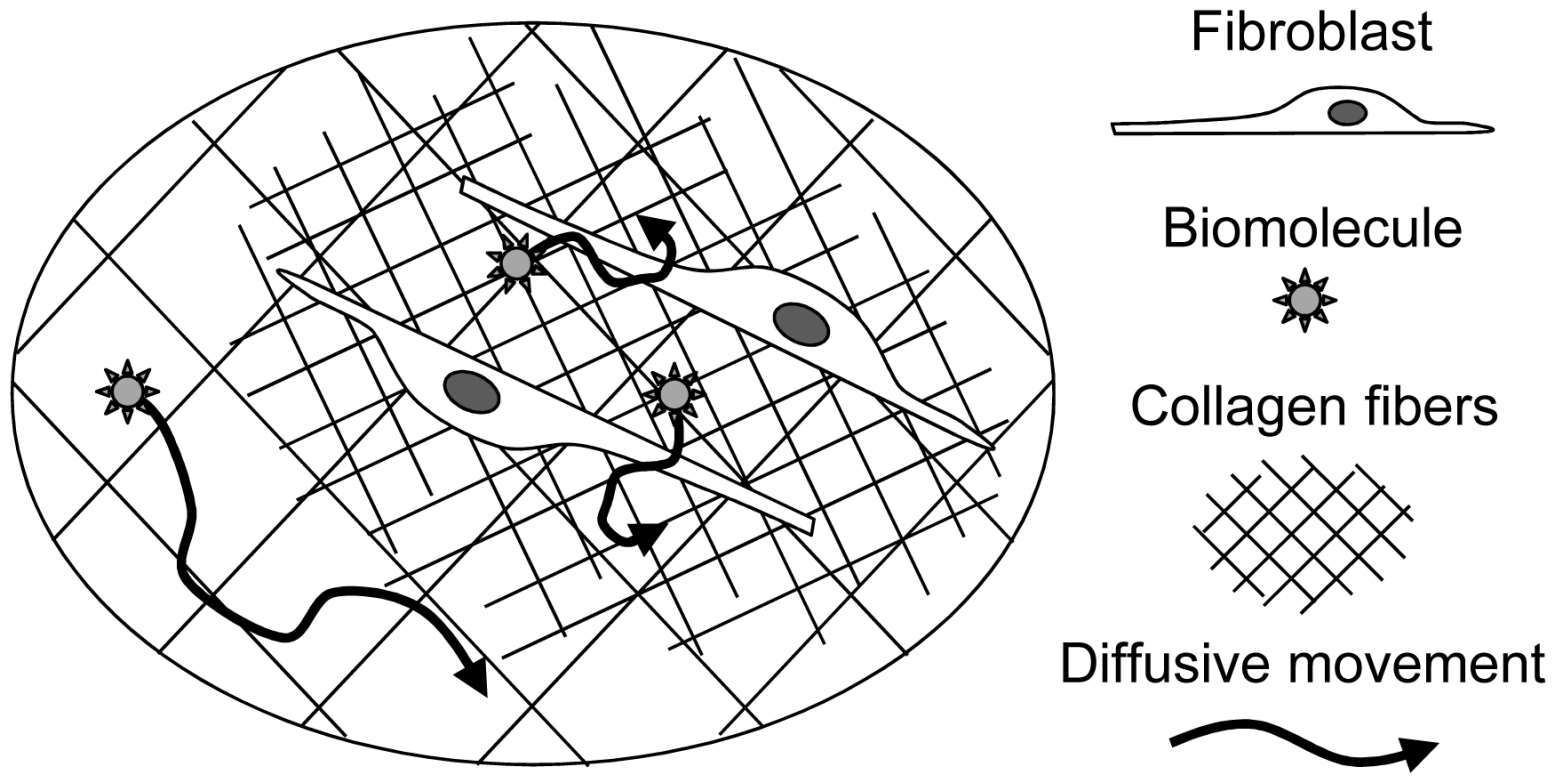
Schematic diagram of probe diffusion in the contracted collagen gel. Collagen fibers are well condensed in the area surrounding the cell due to cellular activity. Biomolecules diffuse throughout the collagen fiber-containing space, in which the concentration of the collagen fibers varies from region to region. Diffusion behaviors of the biomolecules are probably affected by the local concentration of the collagen fibers. By measuring the diffusion behaviors of the biomolecules locally, we can develop a better understanding of the behaviors of these molecules in the heterogeneous ECM and of the physical environment surrounding the cells.

In this study, we measured the diffusion coefficients of size-varied biomolecules in fibroblast-contracted collagen gel by FCS. Since the collagen fibers are condensed into the area surrounding the fibroblasts due to the activities of these cells [13], the sampling space specificity and short measurement time of FCS are suitable for measurement of biomolecular diffusion in the condensed collagen fibers surrounding the cells (Fig. 1). By measuring the diffusion coefficients of size-varied biomolecules, we found features of the physical environment surrounding cells grown in the 3-dimensional collagen fiber environment.

## Materials and Methods

### Materials

Human diploid fetal lung fibroblasts (TIG-1 cells), established at the Tokyo Metropolitan Institute of Gerontology [25], were obtained from the Health Science Research Resources Bank (Osaka, Japan). Type I collagen (acid extraction from porcine tendon) solution was purchased from Nitta Gelatin Co. (Osaka, Japan). Dulbecco's modified Eagle medium (DMEM) and green fluorescence protein (GFP) were purchased from Wako Pure Chemical Industries Ltd. (Osaka, Japan). Fluorescein isothiocyanate (FITC)-labeled dextrans (MW: 4 kDa and 40 kDa) were purchased from Sigma-Aldrich (St. Louis, MO, USA). Fetal bovine serum (FBS) was purchased from JRH Biosciences (Lenexa, KS, USA). Antibiotics, Alexa Fluor 488 (Alexa488)-labeled dextrans (MW: 3 kDa and 10 kDa), Alexa488-labeled alkyne (MW: 774 Da), Alexa488-labeled streptavidin, and Alexa488-labeled IgG were purchased from Life Technologies Japan Ltd. (Tokyo, Japan). FITC-labeled type I collagen was purchased from the Collagen Research Center (Tokyo, Japan). Glass-based culture plates and dishes were purchased from Asahi Glass Co., Ltd (Tokyo, Japan). Other reagents were purchased from Wako Pure Chemical Industries Ltd., Sigma-Aldrich, or Life Technologies Japan Ltd.

### Cell culture

TIG-1 cells from the 35th to 40th population-doubling level were used. The cells were maintained in DMEM containing 10% FBS and antibiotics (100 units/mL penicillin G and 100 µg/mL streptomycin sulfate) at 37°C under a humidified atmosphere of 5% CO_2_ and 95% air. The culture medium was replaced twice a week.

### Preparation of collagen gels containing cells

TIG-1 cells were removed from dishes by treatment with 0.25% trypsin-0.02% EDTA in PBS, washed with the culture medium, counted using a hemocytometer, and suspended at an appropriate density. Collagen solution containing TIG-1 cells in the culture medium was prepared by mixing collagen solution (3 mg/mL) with 5× concentrated DMEM, FBS, and cell suspension at 4°C to give a final collagen concentration of 2.1 mg/mL and a final cell density of 2.5×10^5^ cells/mL. The prepared cell-containing collagen solution was plated on a glass-based plate, and the plate was incubated for 1 h at 37°C for gelation before the addition of the culture medium.

### CLSM

Collagen gels constructed with 0.7 mg/mL FITC-labeled type I collagen populated with TIG-1 cells on glass-based dishes were observed by CLSM (FV-1000D; Olympus, Tokyo, Japan) equipped with a 60× water immersion lens (NA = 1.2).

### FCS measurement and analysis

After 3 days of culture, fluorescent dyes in the culture media were applied to the collagen gels with or without TIG-1 cells and incubated at 37°C for 3 h. After incubation, the specimens were left at 32°C for 30 min. FCS measurement was performed using a confocal laser scanning microscope equipped with a 60× water immersion lens at 32°C. All fluorescent dyes were excited by a 473-nm diode laser. Unless otherwise indicated, the measurement points of the collagen gel with TIG-1 cells were in the extracellular space with 500 nm of the cell surface. The heights of the all measurements were fixed. The fluorescence intensity was measured in the photon-counting mode, and the scan speed and scanning time were set at 2 µs/pixel and 32766 times, respectively.

The acquired single data were divided into 64 parts, and the datasets were analyzed by software supplied by Olympus with a fitting program. Each diffusion coefficient was represented by the average of the 64 parts of fitting values, and we measured the diffusion coefficient more than 10 times while changing positions using single or double collagen gels in each condition.

The autocorrelation curve was obtained by correlating the fluorescence intensity trace shift within a given time interval. The time shift *τ* was varied, and the correlation curve was obtained by multiplying the deviation of the average intensity, *I*, at the time point *t* with the deviation at time point *t+τ* and averaging over the whole trace. Finally, the correlation function, *G(τ)*, was normalized to the squared average signal.
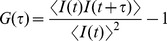
(1)


The diffusion of one single component is commonly fitted with the standard model [26]:



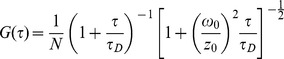
(2).

The resulting ideal probe volume was approximated by a Gaussian profile with the extension *ω_0_* in *x* and *y* directions and *z_0_* in the *z* direction [27]. *N* is the number of fluorescent molecules in the detection volume, defined by a radius *ω_0_* and a length *2 z_0_*. The diffusion time (*τ_D_*) was defined as the lateral diffusion time for a molecule through the volume,



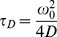
(3).

The diffusion coefficient of GFP at 23°C, 87 µm^2^/s [28], [29], was used as an authentic value for determination of the *ω_0_* and *z_0_*.

The diffusion of spherical molecules was related to various physical parameters by the Stokes-Einstein equation as follows:
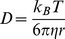
(4)where *T* is the absolute temperature, *r* is the radius of the spherical molecule, *η* is the fluid-phase viscosity of the solvent at 32°C, 0.000768 Pa·s, and *k_B_* is the Boltzman constant.

### Estimation of collagen fiber rearrangement

The condensation of collagen fibers was calculated as a structure model proposed by Ogston et al. [30], [31] describing the diffusion of macromolecules through an array of straight cylindrical fibers of radius *r_f_* and fiber volume fraction *Φ*:
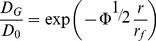
(5)where *D_0_* is the diffusion coefficient in solution, *D_G_* is the diffusion coefficient in the gel, and *r* is the radius of the spherical molecule. The initial volume fraction of collagen gel fibers *Φ_i_* was 2.1×10^−3^. When the affected radius of cylindrical fibers *r_f_* for each fluorescent dye was considered to be constant, the estimated condensation of collagen gel fibers (*Φ_c_/Φ_i_*) was calculated by



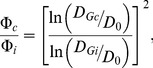
(6)where *Φ_c_* is contracted volume fraction of collagen gel fibers, *D_Gc_* is the diffusion coefficient in the contracted gel, and *D_Gi_* is the diffusion coefficient in the initial gel.

### Statistical analysis

The results of the experiments are expressed as means ± standard deviations. The mean values for each group were compared by analysis of variance followed by Welch's *t*-test. *p*-values of less than 0.01 were considered statistically significant.

## Results

### Collagen gel contraction by TIG-1 cells

**Figure 2:**
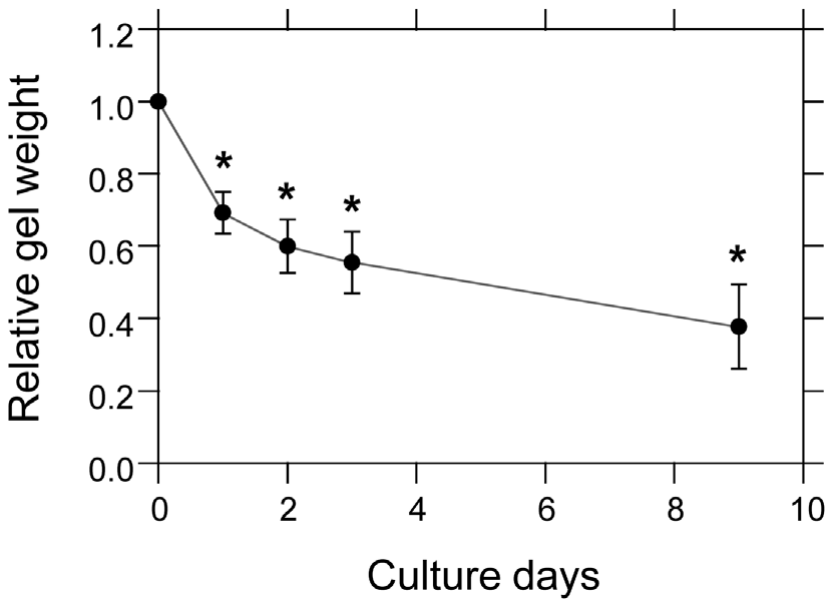
The contraction process of the cell-populated collagen gel. Gel contraction was evaluated by measuring gel weight. One milliliter of collagen solution including 2.5×10^5^ TIG-1 cells was plated on a 35-mm culture dish and gelated. After gelation, the cell-populated collagen gel was cultured. The collagen gel weight was measured after removing culture medium from the dish. Each point is the mean ± standard deviation (n = 4). * *p*<0.01 vs. initial collagen gel weight.

First, we evaluated the process of collagen gel contraction, which we used as a model for the dynamic steps of cell-dependent construction of the heterogeneous environment of the ECM. In this study, in order to allow for measurement of the contracted collagen gel by CLSM, we used collagen gels tightly attached to the surface of glass-based culture dishes or plates. Then, we evaluated the gel contraction process macroscopically by determining the weight of the collagen gel (Fig. 2). The initial collagen content in the gel was only 0.21% (w/v), and thus almost all of the gel contained culture media. Therefore, the gel contraction process could be simply evaluated by measuring the change in the weight of the collagen gel in the culture dish. The collagen gel weight decreased with culture time, and after 9 days in culture, the weight of the gel decreased to approximately 0.4-fold of its original weight (Fig. 2). Rapid contraction occurred during the first 24 h of culture, and this process was same as previous gel contraction study [32]. The collagen gel was macroscopically contracted by about 1.8-fold after 3 days of culture with TIG-1 cell.

**Figure 3:**
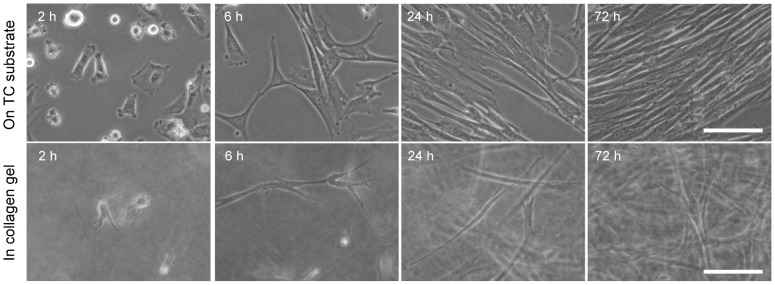
Phase contrast micrographs of TIG-1 cells. TIG-1 cells were cultured on regular tissue culture (TC) substrate or in collagen gel. The cell suspension in the culture medium or collagen solution was plated on a 35-mm culture dish. Each time is shown at the upper left of the corresponding micrograph. Bar: 100 µm.

**Figure 4:**
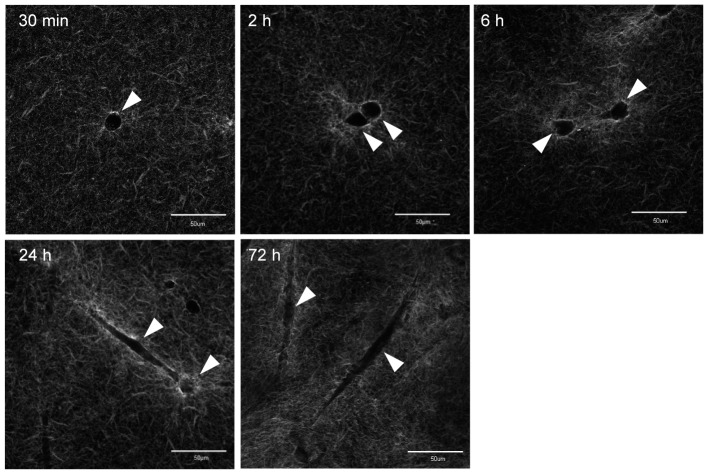
Fluorescent micrographs of the condensation process of collagen fibrils by populated fibroblasts. TIG-1 cells were cultured in FITC-labeled collagen gels (0.7 mg/mL). The populated cells were seen as completely black objects (arrowheads). Each time is shown at the upper left of the corresponding micrograph. FITC-labeled collagen fibers were condensed into the area surrounding the cell. Bar: 50 µm.

TIG-1 cells show unique morphological changes in the process of collagen gel contraction [13], [33]. Although TIG-1 cells adhered and spread well on normal culture dishes, when grown in collagen gel, they exhibited a spherical shape, with projections at 2 h of culture, and gradually elongated to exhibit a spindle-type morphology from 6 h of culture in the collagen gel (Fig. 3). We then examined the timing and conditions of rearrangement of the collagen fibers with TIG-1 cells by culturing cells in collagen gel comprised of FITC-labeled type I collagen by CLSM. This method enabled us to visualize the rearrangement process of collagen fibers in the collagen gel [13]. Fluorescent fibers containing FITC-labeled type I collagen molecules began to be condensed around the round-shaped cells with projections at 2 h of culture (Fig. 4). The fibers were condensed radially within the area surrounding the cell body. After 24 h, the collagen fibers were condensed around the elongated cell body as well as the pseudopodia. Although the rapid changes in cell morphology and collagen gel contraction did not begin in the first several hours of collage gel culture [13], [32], the cells began to cause condensation of the collagen fibers by elongation and retraction of their projections (Fig. 4). TIG-1 cells physical interacted with and rearranged the collagen fibers, thereby organizing the heterogeneous environment of the ECM.

### Diffusion coefficients of biomolecules in the collagen gel

**Figure 5:**
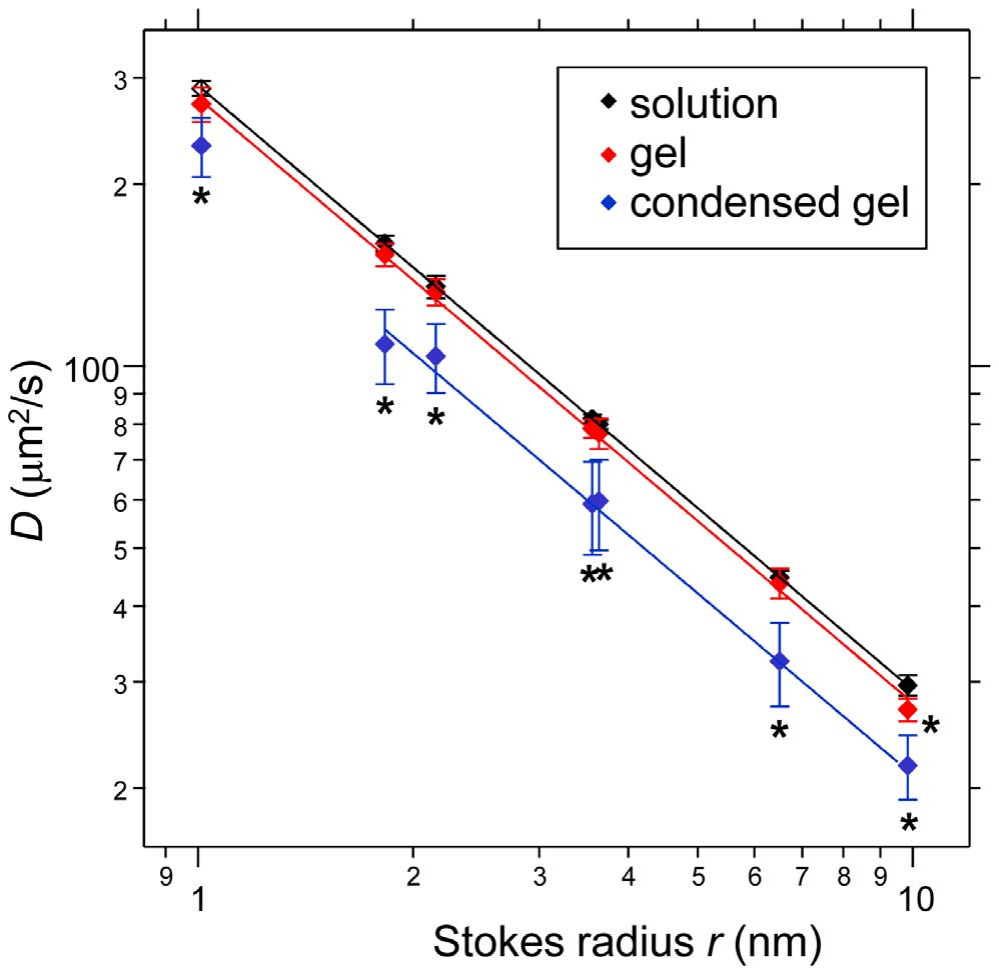
Diffusion coefficients of biomolecules in each condition. The diffusion coefficients (*D*) of biomolecules (Alexa488 alkyne, Alexa488 dextran [3 kDa], FITC dextran [4 kDa], Alexa488 dextran [10 kDa], FITC dextran [40 kDa], Alexa488 streptavidin, and Alexa488 IgG) were measured by FCS in the solution, collagen gel (2.1 mg/mL), or cell populated and condensed collagen gel. In the condensed collagen gel, the *D* of the biomolecules was measured at the surface of cells (∼500 nm). Each *D* was plotted versus each molecular Stokes radius, *r*. Each point is the mean ± standard deviation (n = 10 or 20). The lines show the inverse correlation between *D* and *r*. * *p*<0.01 vs. *D* in the solution of the same biomolecule.

The diffusion coefficient in culture media, *D_0_*, was measured for each biomolecule by our FCS system. The Stokes radius (*r*) was calculated with Eq. (4) from the determined diffusion coefficient *D_0_* (Table 1). The ranges of the Stokes radii were approximately from 1 to 10 nm, and thus the probe set covered the radii of low molecular weight compounds and proteins. Next, we measured the diffusion coefficient of each biomolecule in collagen gel with or without cells. In the cell-populated contracted gel, we measured the diffusion coefficient of the probes in the vicinity of the cells (∼500 nm) at 3 days of culture. The diffusion coefficients in control collagen gel slightly decreased as compared to those in solution (Fig. 5). On the other hand, those in the contracted gel varied widely and were largely decreased compared with those in solution. Therefore, the diffusion coefficients of the differently sized probes were apparently affected by the contracted gel environment.

**Table 1:** Diffusion coefficients and Stokes radii of the probes.

	Diffusion coefficient	Stokes radius
	*D_0_* (µm^2^/s)	*r* (nm)
Alexa488 alkyne	288±8	1.01±0.03
Alexa488 dextran [3 kDa]	160±5	1.82±0.05
FITC dextran [4 kDa]	135±6	2.15±0.09
Alexa488 dextran [10 kDa]	81.7±1.4	3.56±0.06
FITC dextran [40 kDa]	44.7±1.1	6.52±0.16
Alexa488 streptavidin	80.1±1.3	3.64±0.06
Alexa488 IgG	29.6±1.2	9.84±0.39

**Figure 6:**
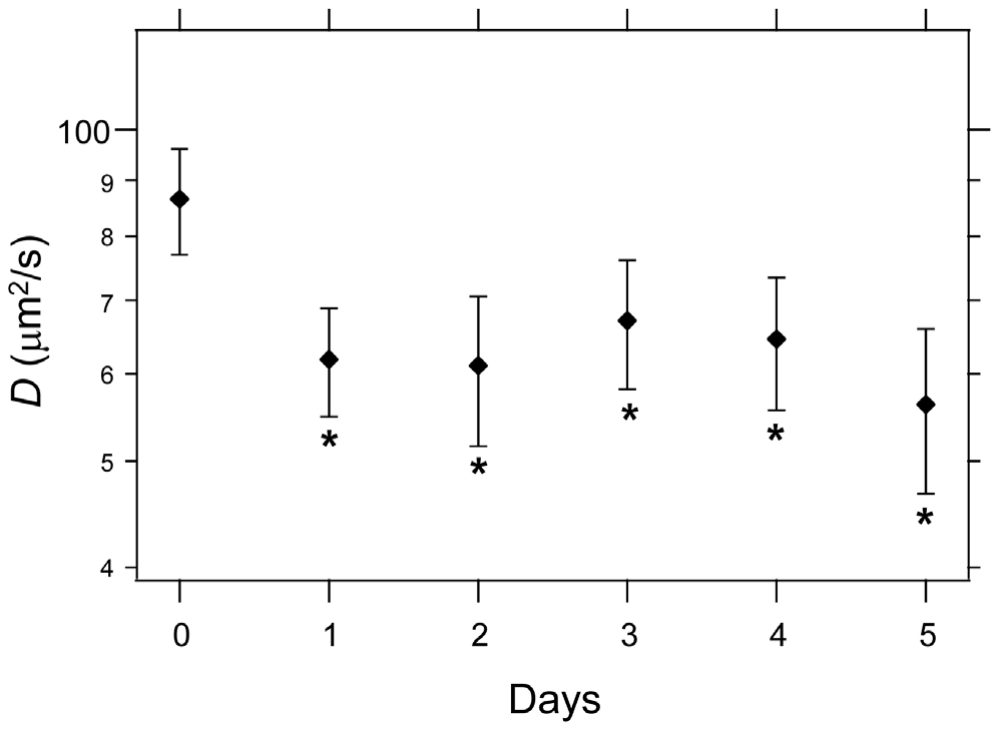
Changes in biomolecular diffusion during the culture of cells in collagen gel. The *D* of Alexa488 dextran (10 kDa) was measured at the surface of the cells (∼500 nm) on each culture day. The data for day 0 was that of cells incubated with the collagen gel for 30 min. Each point is the mean ± standard deviation (n = 20). * *p*<0.01 vs. *D* at day 0.

Since our data supported that molecular diffusion was influenced by the contracted gel environment, we then examined the temporal transition of molecular diffusion during gel contraction. The diffusion coefficient *D* of Alexa488-labeled dextran (10 kDa) in the vicinity of the cells was measured every 24 h (Fig. 6). The diffusion coefficient on day 0 was considered that of molecules after a 30-min incubation of the cell-populated collagen gel. The diffusion coefficient in the cell-populated collagen gel clearly decreased (to 0.71-fold that of day 0) in the first 24 h culture, but only exhibited minor changes for the remaining experimental period. This transition process of the diffusion coefficient in the cell vicinity was similar to that of macroscopically observed rapid gel contraction within the first 24 h (Fig. 2). After the first 24 h, we could not judge the validity of correlations between biomolecular diffusion and macroscopically observed gel contraction, which was a gradual decrease with time. Condensation of the FITC-labeled collagen fibers within the area surrounding the cells began in the first several hours of culture and was fully induced within the first 24 h culture (Fig. 4). Thus, the diffusion coefficient of biomolecules was influenced by the cell surrounding local space.

**Figure 7:**
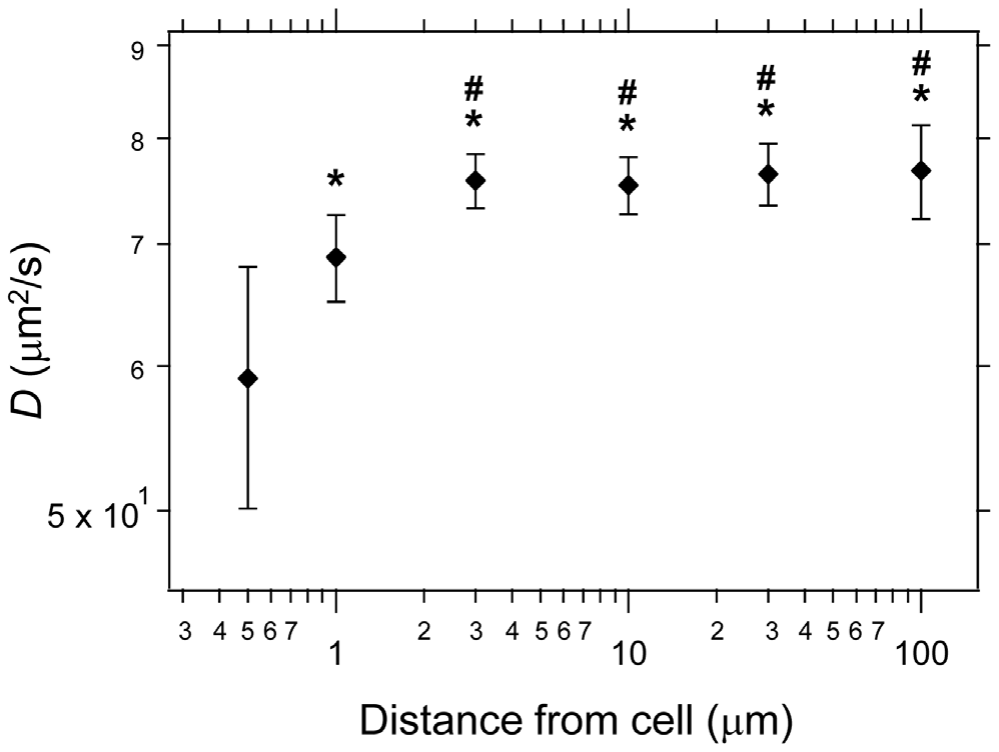
Diffusion coefficients of biomolecules in the space surrounding the gel-containing cell surface. The *D* of Alexa488 dextran (10 kDa) was measured at various distances from the cell surface of cells grown in the contracted gel at 3 days of culture. Each point is mean ± standard deviation (n = 10). * *p*<0.01 vs. *D* at the distance of 0.5 µm from the cell. **#** *p*<0.01 vs. *D* at the distance of 1.0 µm from the cell.

Then, to investigate whether the diffusion coefficient reflected the condensation of collagen fibers, we measured that of Alexa488-labeled dextran (10 kDa), in which the measuring position was shifted from the cell edge (Fig. 7). The diffusion coefficient increased with increasing distance from the cell, and was almost constant when the distance was more than 3 µm from the cell. That is, the biomolecular diffusion of molecules with a 3.56-nm radius was influenced by the local environment of the extracellular space. Molecular diffusion was dramatically restricted in the vicinity of the cell, and the inhomogeneous physical environment of the extracellular space was constructed by cellular activity.

### Inference of collagen fiber rearrangements by fibroblasts

**Figure 8:**
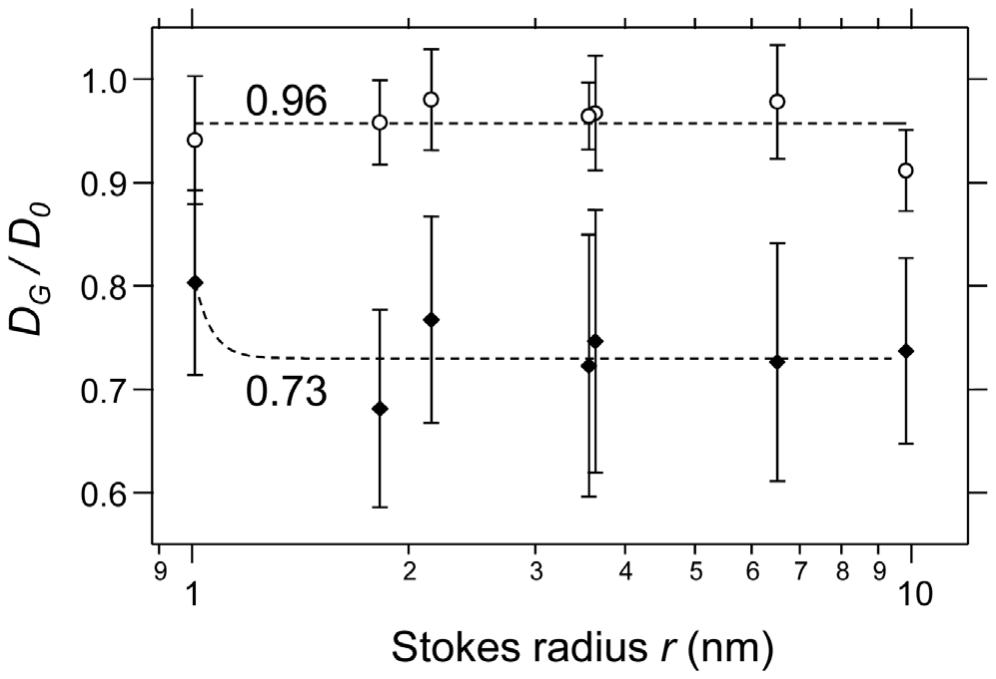
Relative values of diffusion coefficients in collagen gel and in solution. The relative values of diffusion coefficients in collagen gel (*D_G_*) compared to those in solution (*D_0_*) for each biomolecule are plotted versus the Stokes radius, *r*. Each *D* was already plotted in Fig. 5. The open circles show the relative data of the normal collagen gel (2.1 mg/mL; *D_Gi_/D_0_*), and the closed diamonds show the relative data of the contracted collagen gel (*D_Gc_/D_0_*). Each point is the mean ± standard deviation (n = 10 or 20).

Finally, we evaluated the rearrangements of collagen fibers by TIG-1 cells using the results of diffusion coefficients of the molecules (Fig. 5) with the Ogston structure model [30], [31]. The Ogston model is a stochastic model for diffusion of spherical particles in an array of fibers. Fig. 8 shows the *D_G_/D_0_* of each probe in the contracted gel or the control collagen gel at 3 days of culture. The average value of the *D_Gi_/D_0_* in control collagen gel was 0.96. On the other hand, the *D_Gc_/D_0_* values of probes ranging from 1.8 to 10 nm in size were widely distributed, with an average value of 0.73. From Eq. (6), using these *D_G_/D_0_* values and the initial volume fraction of collagen gel fibers *Φ_i_* (approximately 2.1×10^−3^), the volume fraction of condensed collagen gel fibers *Φ_c_* was 1.1×10^−1^ (≈1.1×10^2^ mg/mL). That is, based on the diffusion coefficients of the probes in the collagen gel, the hypothetical collagen fibers were condensed about 52-fold. Therefore, molecular diffusion provided an efficient indicator of the characteristics of the physical environment within the extracellular space.

## Discussion

This study is the first report of the diffusion behavior of molecules in the ECM surrounding cells. Our data demonstrated that the ECM was dynamically constructed and rearranged by the cells via reorganization of the matrix structure. Cells populating 3D matrices (e.g., type I collagen gel) have been shown to exhibit *in vivo*-like behaviors [11], [18], [34]. Thus, in this study, using fibroblast-mediated collagen gel contraction, we evaluated the dynamic diffusion of biomolecules ranging in size from about 1 to 10 nm. This range of biomolecular radii encompasses the sizes of small compounds to large proteins, which are secreted by cells and are often transferred by diffusion throughout the extracellular space. For example, secreted cytokines move through the extracellular space and affect target cell signaling pathway via autocrine and paracrine signaling. Therefore, the diffusion coefficient of each molecule in the extracellular space is a fundamental physical measure of its ability to influence chemical reactions and associated receptors.

In Newtonian fluid solutions, molecules diffuse according to their molecular radius and the temperature and viscosity of the solution [Eq. (4)]. When the temperature and viscosity are constant, the diffusion coefficient *D* is inversely proportional to the molecular radius *r*. In the noncontracted original collagen gel, the diffusion coefficient (*D_Gi_*) was almost the same as that in solution and was completely inversely proportional to *r*. On the other hand, in the fibroblast-containing contracted collagen gel, the diffusion coefficients (*D_Gc_*) in the vicinity of the cells decreased and were approximately inversely proportional to *r*, with the exception of that of Alexa488 alkyne (where *r*≈1 nm). Therefore, biomolecules with radii of about 1.8–10 nm diffuse in the vicinity of cells in contracted collagen gels similar to how they would diffuse in more viscous Newtonian fluid (about 1.34-fold more viscous than water). The *in vitro* reconstituted type I collagen fibers form gel-like structures, and these gel fibers create a complex network structure. Thus, in a theoretical sense, as shown by Monte Carlo simulation of molecular diffusion in gels, molecules, which have only negligible interactions with the gel fibers themselves, diffuse anomalously depending on their size [35]. Small molecules (*R/R*_∞_<0.2; *R* is tracer size, and *R*_∞_ is the minimum size of a trapped tracer ≈ pore size of gel network) diffuse freely in gels and are only minimally affected by the gel network. However, in an actual hydrogel, even in the case of the small tracer (*R/R*_∞_<0.05) with negligible interaction with fibers, the diffusion coefficient decreases in agarose gel, while the *D_g_/D_0_* is almost constant [36]. The authors assumed that the reason for the difference from the Monte Carlo simulation depended on the nonspecific van der Waals interactions between diffusing particles and the polymer network. Reconstituted type I collagen fibers have diverse diameters (average of about 100 nm) with many branches [37]. The contracted collagen gel contains highly complex networks of fibers with diverse radii [13]. Thus, it is possible that the biomolecules in the ECM surrounding cells interact nonspecifically via van der Waals interactions with condensed collagen fibers and then diffuse throughout the collagen fibers similar to diffusion in viscous Newtonian fluid.

The FITC-labeled collagen gel (0.7 mg/mL) condensed around the cells, and the region 10–20 µm around the cells contained particularly well-condensed FITC-labeled collagen fibers. In contrast, a study using scanning electron microscopy showed that the range of the distance of highly condensed collagen fibers is about 1 µm [13]. Our result of the diffusion coefficient of Alexa488 dextran (10 kDa) in the contracted gel showed that diffusion within 1 µm from a cell was restricted and was almost constant when the distance was more than 3 µm from the cell. Therefore, collagen fibers are highly condensed in the cell vicinity and the diffusion of biomolecules is strongly affected by the physical environment constructed from the highly condensed collagen fibers.

In this study, using the diffusion coefficients of the fluorescent probes, we calculated that collagen fibers were hypothetically rearranged and condensed by TIG-1 cells by approximately 52-fold (or 1.1×10^2^ mg/mL) in the vicinity of the cells. This value was higher than that of our macroscopic observation of collagen condensation. Although the estimated value of fibroblast-mediated collagen condensation varied in experimental conditions (e.g., cell number, cell population doubling level, initial collagen concentration, and culture supplements) [11], [18], [33], the density of the fully contracted collagen gel has been shown to reach about 15 mg/mL collagen [16], [17]. This estimated value is the macroscopic density of the contracted collagen gel. Our calculated collagen fiber condensation value could be considered as the effective value of the physicochemical environment for biomolecules with a size range of 1.8 to 10 nm in the ECM surrounding the cell.

As compared to *in vivo* tissue, the extracellular environment of *in vitro* contracted collagen gel is different. Collagen fibers in the mature tissue run along fibroblasts, and glycosaminoglycans are distributed within the space among collagen fibers. On the other hand, in the contracted collagen gel, the collagen fibers are not aligned [13], and probably distribution of glycosminoglycans is insufficient within the gel. Glycosaminoglycans can function as a traveling barrier for cationic biomolecules due to their charge. However, the effects of highly concentrated hyaluronan on the diffusion coefficient of Alexa fluor 488 are very small (∼5%) [3]. Thus, the physical effects of highly condensed collagen fibers on diffusive behaviors of biomolecules shown in this study are larger. In addition, collagen gel contraction is an *in vitro* model of organogenesis or wound contraction [18]. Therefore, our findings of the diffusion behavior of biomolecules in the extracellular space provide a fundamental understanding of the dynamic physical environment of the ECM.

In conclusion, our study demonstrated that biomolecular diffusion in the cell vicinity was strongly affected by collagen fiber rearrangement and condensation, caused by the cells. Biomolecules with sizes of less than 10 nm diffused in the ECM surrounding the cell as though they were diffusing through a more viscous Newtonian fluid. Using the calculated diffusion coefficients of the biomolecules, we estimated the condensation of collagen fibers in the region surrounding the cells. By clarifying the diffusion coefficients of biomolecules having different sizes in the ECM surrounding the cell, we can improve our knowledge of the actual physicochemical environment of the extracellular space, which affects the activity of biomolecules.
